# Moderate or severe scorpion sting: identification of risk factors

**DOI:** 10.1590/1980-220X-REEUSP-2023-0022en

**Published:** 2023-10-27

**Authors:** Carina Akemi Takehara, José Luiz Tatagiba Lamas, Renata Cristina Gasparino, Suzimar de Fátima Benato Fusco

**Affiliations:** 1 Universidade Estadual de Campinas, Faculdade de Enfermagem, Campinas, SP, Brazil.

**Keywords:** Scorpion Stings, Risk Factors, Nursing, Picaduras de Escorpión, Factores de Riesgo, Enfermería, Picadas de Escorpião, Fatores de Risco, Enfermagem

## Abstract

**Objective:**

To characterize scorpion accidents at the Information and Toxicological Assistance Center (CIATox) in Campinas, to analyze risk factors related to the moderate and severe classification, and to determine the age group at greatest risk for this classification.

**Method:**

Cross-sectional and retrospective study, with patients assisted in person at CIATox, who had a scorpion accident, from January 2015 to December 2019. Descriptive and inferential analysis was conducted. For the age variable, a ROC curve was constructed to determine cutoff points in relation to the severity classification. Poisson regression models were adjusted considering severity classification as the dependent variable.

**Results:**

A total of 754 cases with a mean age of 36.05 years, mostly female and non-occupational accidents that occurred in the urban area, was analyzed. The most frequent scorpion was the *Tityus serrulatus*. The risk factors found for greater severity were age group up to 22 years and previous care in other health services.

**Conclusion:**

The age range up to 22 years old should be used as a predictive factor of severity in the clinical evaluation of patients stung by scorpions to carry out adequate management of cases.

## RESUMO

**Objetivo:**

Caracterizar os acidentes escorpiônicos do Centro de Informação e Assistência Toxicológica (CIATox) de Campinas, analisar fatores de risco relacionados à classificação moderada e grave e determinar faixa etária de maior risco para esta classificação.

**Método:**

Estudo transversal e retrospectivo, com pacientes atendidos presencialmente pelo CIATox, que sofreram acidente escorpiônico, de janeiro de 2015 a dezembro de 2019. Foi realizada análise descritiva e inferencial. Para a variável idade foi construída uma curva ROC com o intuito de determinar pontos de corte em relação à classificação de gravidade. Foram ajustados modelos de regressão Poisson considerando a classificação de gravidade como variável dependente.

**Resultados:**

Foram analisados 754 casos com idade média de 36,05 anos, maioria do sexo feminino e acidentes não ocupacionais ocorridos na zona urbana. O escorpião mais frequente foi o *Tityus serrulatus*. Os fatores de risco encontrados para maior gravidade foram faixa etária até 22 anos e atendimento prévio em outros serviços de saúde.

**Conclusão:**

A faixa etária até 22 anos deve ser usada como fator preditivo de gravidade na avaliação clínica dos pacientes picados por escorpião para realização de manejo adequado dos casos.

## RESUMEN

**Objetivo:**

Caracterizar los accidentes de escorpiones en el Centro de Información y Asistencia Toxicológica (CIATox) de Campinas, analizar los factores de riesgo relacionados con la clasificación moderada y severa y determinar el grupo etario de mayor riesgo para esta clasificación.

**Método:**

Estudio transversal y retrospectivo, con pacientes atendidos presencialmente por el CIATox, que sufrieron un accidente de escorpión, desde enero de 2015 a diciembre de 2019. Se realizó un análisis descriptivo e inferencial. Para la variable edad se construyó una curva ROC con el fin de determinar puntos de corte en relación a la clasificación de severidad. Los modelos de regresión de Poisson se ajustaron considerando la clasificación de severidad como variable dependiente.

**Resultados:**

Se analizaron 754 casos con edad media de 36,05 años, en su mayoría mujeres y accidentes no laborales ocurridos en la zona urbana. El escorpión más frecuente fue el *Tityus serrulatus*. Los factores de riesgo encontrados para mayor gravedad fueron el grupo etario hasta 22 años y la atención previa en otros servicios de salud.

**Conclusión:**

El grupo etario hasta los 22 años debe ser utilizado como factor predictivo de severidad en la evaluación clínica de los pacientes picados por escorpiones para realizar un manejo adecuado de los casos.

## INTRODUCTION

Scorpions are venomous animals present in different parts of the world and the clinical importance of their sting is related to its progression, which can present with serious and life-threatening manifestations, such as damage to the myocardium, cardiac arrhythmias, pulmonary edema, and shock^(1-5)^.

The annual number of scorpion stings exceeds 1.2 million, leading to over 3,250 deaths^(5)^. In Brazil, it constitutes a public health problem and, according to data from the Information System for Notifiable Diseases of the Ministry of Health, there was a significant increase in the number of cases from 2007 to 2022^(6)^. Data from 2020 to 2022 show 308,904 reported cases of scorpionism, with 375 deaths related to the scorpion sting. The states of Minas Gerais and São Paulo were the ones that registered the greatest number of notifications^(6)^.

An ecological and retrospective study with secondary data on scorpion stings in the state of São Paulo from 2008 to 2018 analyzed 145,464 cases and found a gradual increase in the incidence of scorpion stings, starting with 14 cases per 100,000 inhabitants-years in 2008 and reaching approximately 70 cases per 100,000 inhabitants-years in 2018, corresponding to an increase of 378.5% in the period^(7)^.

In Brazil, scorpions of clinical importance, which can cause severe envenomation, belong to the family *Buthidae*, genus *Tityus* ^(1-5)^. There is a predominance of the species *Tityus serrulatus* (yellow scorpion), with good adaptation to the urban environment and great parthenogenetic proliferation, being present in virtually all regions, including the Southeast, Midwest and South, with the exception of some states in the North region, and *Tityus bahiensis* (brown scorpion), from the Northeast region and present in Minas Gerais, São Paulo, Paraná and Santa Catarina, with a more limited distribution due to its ecological niche and sexual reproduction^(7,8)^.

The *Tityus serrulatus* sting is responsible for most cases of severe and fatal progression^(3,4,9)^. The toxins released in the sting can cause serious cardiovascular and respiratory effects due to the action of the venom on brainstem and spinal cord structures that act in neurovegetative control^(3,9)^, and massive autonomic response^(4,9)^. The venom, or part of it, can cross the blood-brain barrier, mainly in young individuals, in whom it is not completely formed, reaching the central nervous system. Other authors state that the central effects would be caused by the peripheral stimulation of the venom^(10)^.

The Ministry of Health proposes the classification of severity of scorpion accidents according to clinical manifestations: the accident is considered mild if it only presents local pain, with or without paresthesia; moderate, if there is severe local pain and systemic manifestations (slight sweating, nausea, occasional vomiting, tachycardia, tachypnea, and hypertension); if, in addition to the moderate form, there is profuse and uncontrollable vomiting, profuse sweating, intense sialorrhea, prostration, seizures, coma, bradycardia, heart failure, severe pulmonary edema and shock, the cases are classified as severe. Moderate and severe cases should be treated with serum therapy^(11)^.

Another taxonomy used to compare scorpion stings within different countries is that of Khattabi, developed by an international consensus of experts, which assesses the outcome of scorpion stings^(12)^. This classification divides the cases into dry sting (without envenomation), class I (local manifestations only), class II (non-life-threatening clinical manifestations), class III (life-threatening systemic manifestations - respiratory failure, pulmonary edema, cardiogenic shock, and brain damage) and fatal outcome^(12)^.

The predictors of severity described by the Ministry of Health (MS) are the species and size of the scorpion, the amount of venom inoculated, the victim’s body mass, and the patient’s sensitivity to the venom. Moderate and severe forms are reported to be more frequent in children^(11)^.

In addition, there are other risk factors, such as early diagnosis, time elapsed between the sting and serum administration^(2,11)^, children under 7 years old, and elderly^(2,9,13)^. However, healthy adults are not exempt from progressing to severe cases and deaths^(2)^.

There are Brazilian studies analyzing clinical and epidemiological aspects of scorpionism, such as the one carried out in Minas Gerais, where *Tityus serrulatus* ^(13)^ predominates and there was no statistical difference between the sexes of the individuals affected, and the age group between 55 and 64 years was the one presenting the highest risk for scorpionism. In the Southeast region, a study was carried out in the metropolitan region of Campinas, with patients assisted by the Center for Information and Toxicological Assistance of Campinas (CIATox of Campinas), which analyzed scorpion accidents in the period from 1994 to 2011. There was an increase in the number of cases attended, which progressed only with local manifestations (class I). All severe cases (classified as III or fatal) occurred in children under 15 years associated with *Tityus serrulatus* sting. These cases were treated with anti-scorpion venom serum^(4)^.

A study carried out in Bahia evaluated 3,565 cases of scorpionism, of which 15.9% were classified as more severe, associated with the age groups from 0 to 19 years and 60 or more, and the time elapsed between the moment of the sting and the hospital care of more than 3 hours^(14)^. Another study, conducted in the region of Santarém (Pará), revealed that most victims were men (83.3%) with a mean age of 33.6 +/– 18.3 years and length of medical care of 4.6 +/– 3.2 hours. Local symptoms occurred in 91.7% of the cases and systemic manifestations in 98.6% of the accidents. Despite only 8.3% of identification, all were of the species *Tityus cambridgei,* presenting neurological manifestations, which is particularly different from envenomations caused by other species of scorpions present in Brazil^(15)^. Research with data from 2007 to 2017 in the state of Alagoas, Northeastern Brazil, in a referral hospital for the care and treatment of accidents by venomous animals, analyzed 27,988 cases, and observed a higher frequency of scorpion stings in women, age group affected from 20 to 29 years old, significantly associating the occurrence of systemic manifestations and the severity of the cases to pediatric patients up to 4 years old (69.4%) and 50% of the total cases treated with serum therapy corresponded to patients in this age group^(16)^.

Thus, considering the heterogeneity of the epidemiological distribution and the risk factors associated with the severity of scorpionism, it is essential to study the characteristics of scorpion accidents related to the region covered by CIATox Campinas/SP so that assistance can be increasingly targeted and severe cases prevented.

Therefore, the objective is to characterize the scorpion accidents at CIATox in Campinas, to analyze risk factors related to the moderate and severe classification, and to determine the age group at greatest risk for this classification.

## METHOD

### Design of Study, Local and Population

This is a descriptive, cross-sectional, and retrospective study, carried out with all patients who suffered a scorpion accident and were attended in person by CIATox in Campinas at the Hospital de Clínicas da Unicamp (HC), from January 1, 2015 to December 31, 2019. It is important to highlight that all patients who seek urgent and emergency care at HC due to a scorpion accident are attended to in person by CIATox’s on-duty physicians, as well as those primarily assisted in other services and referred to CIATox because were classified as moderate or severe cases.

### Data Collection

Data were collected from electronic attendance records by the researchers from September 2020 to February 2021. The following were collected: name initials, medical record number, age, type of occupational/accidental accident, neighborhood/subdistrict, city, state of residence, day, month, year and time of care, day of the scorpion accident, time until the beginning of the service at CIATox, identification/species of scorpion, anatomical region of the sting, treatments performed at other services (called previous treatment) and directly at CIATox, symptoms and signs presented, and classification of the accident according to the Ministry of Health^(11)^ and Khattabi et al.^(12)^.

### Data Analysis and Treatment

After data collection, they were entered directly into an Excel database. All data were double-checked and inconsistencies were corrected. Descriptive and inferential analysis was performed using the Mann-Whitney and Chi-square tests^(17)^, as relevant. For cases in which the assumptions of the Chi-square test were not met, Fisher’s exact test was applied^(18)^. Modified multiple Poisson regression models were adjusted, with robust variance, considering the classification of severity as the dependent variable^(19)^. The results show the estimates obtained for the prevalence ratio, as well as their respective confidence intervals and p-values.

For the age variable, the distribution of moderate and severe cases was separated by age group up to 14 years old, according to data presented in the Notifiable Diseases Information System (*SINAN*), to allow comparisons. A ROC curve was also constructed to determine cutoff points in relation to the severity classification. The results of the ROC Curve were described through the graphical representation of the curve and the area under the curve with its respective confidence interval. The area under the curve represents the ability of the test to discriminate individuals with or without the outcome. Values greater than or equal to 0.70 can be considered adequate^(20)^.

A first cutoff point was established according to the highest Youden et al.^(21)^ index, calculated by deducting 1 from the sum of sensitivity and specificity of the test and not expressed as a percentage, but as part of an integer: (sensitivity + specificity) – 1. The second cutoff point was determined considering the milestone where sensitivity was equal to specificity. For the analyses to be carried out, a significance level of 5% and the software SAS 9.4 and SPSS 23 were considered.

### Ethical Aspects

The research followed the guidelines of Resolution No. 466/2012 of the National Health Council (CNS) for scientific research in human beings, with waiver of presentation of the free and informed consent form, since data were obtained from secondary sources. Confidentiality and anonymity of the participants were ensured. The project was approved by the Research Ethics Committee (CEP) of the Universidade Estadual de Campinas (UNICAMP), under opinion number 4.218.549 of 2020.

## RESULTS

A total of 754 cases of scorpion stings attended in person at CIATox Campinas from January 2015 to December 2019 was analyzed. The patients’ ages were recorded in 753 medical records and ranged from 1 to 87 years (mean 36.05; standard deviation 19.13 years, median 35.0). The characterization of the sample, the accident, and the distribution of scorpionism cases, according to the taxonomies of the Ministry of Health and Khattabi, are presented in Table 1.

**Table 1:** Epidemiological variables related to the patient and the accident and distribution of scorpion accident classifications – Campinas, SP, Brazil, 2021.

Epidemiological variables	N	%
**Age**		
0 to 14 years	106	14.08
15 years or more	647	85.92
**Sex**		
Female	421	55.84
Male	333	44.16
**Accident location**		
Urban	658	87.27
Rural	80	10.61
Not specified	16	2.12
**Type of accident**		
Non-occupational	604	80.11
Occupational	149	19.76
Not specified	1	0.13
**Type of scorpion**		
*Tityus serrulatus*	142	18.83
*Tityus bahiensis*	50	6.63
Not identified	562	75.54
**Classification Ministry of Health**		
Light	710	94.16
Moderate	36	4.77
Severe	8	1.06
**Khattabi Classification**		
Dry sting	20	2.65
Class I	690	91.51
Class II	33	4.38
Class III	10	1.33
Fatal	1	0.13

Observing the epidemiological distribution shown in Table 1, there are correspondences between the two taxonomies: cases classified as mild by the MS correspond to dry stings added to Khattabi class I; MS moderate ones equivalent to class II (Khattabi); and severe cases of MS are represented by class III plus the fatal outcome. Thus, all analyses were performed according to the MS classification.

Table 2 shows the association between the variables related to the patient, the scorpion accident, and the care according to the Ministry of Health’s classification of severity.

**Table 2:** Association between the variables related to the patient, the scorpion accident, and the care according to the Ministry of Health’s classification of severity – Campinas, SP, Brazil, 2021.

Variables	Ministry of Health Classification		p-value
	Mild n (%)	Moderate/Severe n (%)	
**Age**			
0–14 years	76 (10.72)	30 (68.18)	<0.0001*
15 years or more	633 (89.28)	14 (31.82)	
**Type of scorpion**			
*Tityus bahiensis*	49 (6.90)	1(2.27)	0.478*
*Tityus serrulatus*	134 (18.87)	8 (18.18)	
Not identified	527 (74.23)	35 (79.55)	
**Number of stings**			
Single sting	682 (96.06)	39 (88.64)	0.373**
More than one sting	28 (3.94)	5 (11.36)	
**Primary care**			
CIATox	597 (84.08)	11 (25.00)	<0.0001*
Previous care	113 (15.92)	33 (75.00)	
**Previous analgesia**			
No	620 (87.32)	23 (52.27)	<0.0001*
Yes	90 (12.68)	21 (47.73)	
**Administration of AScVS**			
No	710 (100.00)	12 (27.27)	<0.0001**
Yes	0 (0.00)	32 (72.73)	

* p-value obtained through the chi-square test; **p-value obtained using Fisher’s exact test. AScVC: anti-scorpion venom serum.

The average time between the scorpion accident and care at CIATox was 119.84 (144.18) minutes for cases considered moderate/severe and 197.38 (359.07) minutes for mild cases, with no significant statistical difference in this variable according to the severity classification (p = 0.3845).

**Figure 1:**
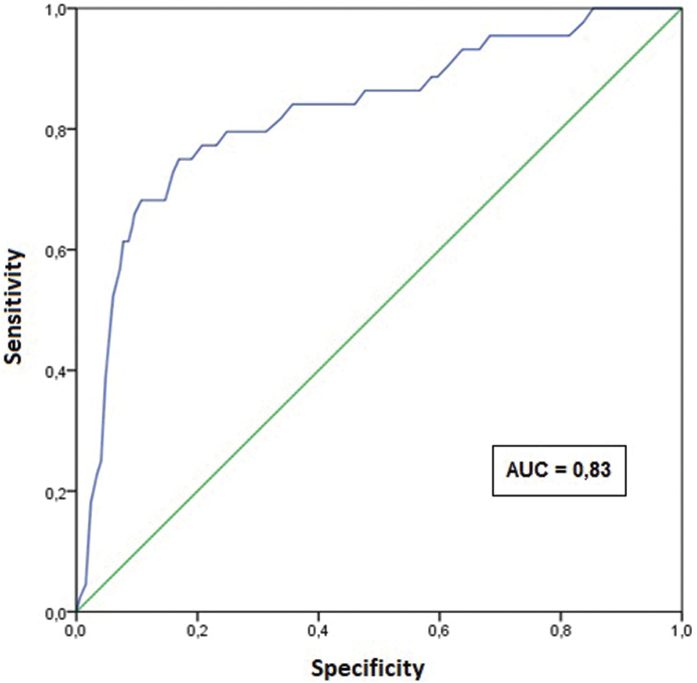
ROC curve: age compared to severity classification – Campinas, SP, Brazil, 2021. AUC: area under the curve.

Figure 1 shows the ROC curve, designed with the aim of establishing a cut-off age for the case outcome to be classified as moderate or severe. We defined two cutoff points using the ROC curve, one from the age of 19, established by the Youden index (0.581), and the other from the age of 22, a milestone where sensitivity was equal to specificity (0.77).

Table 3 shows the prevalence ratio of moderate or severe cases according to age groups defined by the ROC curve.

**Table 3:** Prevalence ratio estimates for moderate and severe cases for models with cutoffs of age groups defined by the ROC curve, according to the accident severity classification of the Ministry of Health (n = 737) – Campinas, SP, Brazil, 2021.

Independent variables	Prevalence ratio*	CI 95%		p-value
		LL	UL	
**Model 1**				
Women	0.89	0.52	1.51	0.6610
Age ≤ 19 years	8.21	4.47	15.10	< 0.0001
Rural accident	1.28	0.73	2.24	0.3945
Occupational cause	1.13	0.35	3.62	0.8342
Previous treatment	8.76	4.72	16.25	< 0.0001
2 or more stings	2.07	0.85	5.06	0.1087
**Model 2**				
Women	0.90	0.54	1.49	0.6684
Age ≤ 22 years	6.40	3.43	11.94	< 0.0001
Rural accident	1.52	0.87	2.67	0.1409
Occupational cause	0.85	0.27	2.69	0.7885
Previous treatment	8.83	4.79	16.26	< 0.0001
2 or more stings	1.98	0.89	4.40	0.0943

p-value obtained through Poisson Regression. CI Confidence Interval. LL lower limit, UL upper limit.

## DISCUSSION

The present work reveals that, for this sample, age equal to or less than 22 years old, as well as previous treatment of symptoms, were considered risk factors for greater severity of scorpion stings.

Data provided by the Notifiable Diseases Information System (SINAN), from 2015 to 2019, the same period of data collection for this study, show the age profile of scorpion stings in Brazil, in which the distribution of moderate and severe cases in the age group up to 14 years was 32.53%; between 15 and 19 years old, 6.18%; and between 20 and 39 years old, 25.56%^(6)^. In the present study, a distribution of 68.18% of this same level of severity was found for age up to 14 years, more than double the relative frequency of this age group notified by SINAN.

Previous study performed in the same CIATox^(4)^, between 1994 and 2011, revealed greater severity of scorpion accidents in the age group corresponding to people under 15 years old, covering 100% of serious or fatal cases. Considering only moderate cases, 201 accidents were recorded, with 126 (62.69%) in patients aged 15 years or older. Our study identified 44 moderate and severe cases, 14 (31.82%) of these cases in patients aged 15 years or older. However, diverging from the previous study, 3 (37.5%) severe cases were found in people aged over 14 years.

A study carried out in Bahia, analyzing 3,565 cases of scorpionism, 13.9% of which were moderate and 2.0% severe, found an association between its severity and the age group of 0 to 9 years, 10 to 19 years, and 60 years or more^(14)^. Epidemiological analysis carried out with 3055 cases in the south of Bahia showed that severe conditions were more frequent among children under 15 years of age^(22)^. Similar research carried out in Turkey, with 201 children between 0 and 17 years old, showed 138 mild, 34 moderate, and 29 severe cases^(23)^. These studies show a divergence in the distribution of more severe cases of scorpion envenomation according to age when compared to the present study, which expands the age range of greatest risk to 22 years.

In our work, the occurrence of previous care by the health service of origin is significantly related to the moderate and severe classification, with an increasing prevalence ratio as the age group increases (0 to 19 years – 8.76; 0 to 22 years – 8.83).

In the initial treatment, some behaviors must be taken with caution, due to specificities of the action of the scorpion venom, and the patient referred as soon as possible to a reference hospital with antiscorpion serum (AScVS) available^(9)^ aiming at the early classification and adequate management of patients.

It was demonstrated that, of the total number of cases that progressed with a moderate and severe classification, 25% were initially cared for directly at CIATox, and 75% received initial care at other services. It should be noted that there was no statistically significant difference between the mean time of the accident and the attendance at CIATox when comparing moderate/severe cases with those considered mild.

These results suggest that the early referral of moderate and severe cases of scorpion envenomation to the reference service in the region, CIATox, indicates a greater capacity of health services of identifying symptoms and signs of severity and referring these patients for adequate monitoring and treatment in a timely manner, which may have contributed to a better clinical progression and reduction of complications resulting from scorpion envenomation.

An association was found between administration of antiscorpion venom serum and moderate and severe cases, with 72.73% of patients with this classification receiving AScVS. This relationship was expected, since the Ministry of Health recommends the use of serum therapy for all cases classified as moderate or severe^(11)^. However, it was possible to note that not all patients initially considered moderate received AScVS, perhaps because they were continuously monitored and followed up by a specialized team at CIATox, from the moment of admission to safe discharge.

However, for the health team to be able to carry out prevention, and act effectively in cases of scorpion stings, they have to be trained, specifically nurses, who are responsible for welcoming patients in primary health care units. This training should aim at the prevention, detection, and comprehensive treatment of patients with rapid and evidence-based interventions, recognizing risk factors and clinical manifestations of severity to reduce complications and mortality. It is also necessary to consider changes in the epidemiological profile of scorpion stings in each region, with the possibility of constant monitoring through computerized systems, bringing quality information, with constant updates and easy access to health teams.

## LIMITATIONS

Regarding the limitations of this study, a discrepancy is identified in the studies in relation to the age ranges for analysis, as they did not indicate the election for the analyzed age groups, which compromised the comparability of results. As this is a retrospective study with analysis of previously completed medical records (secondary source), whose information was collected for the purpose of clinical care and not research, the data collected may partially impair information quality. A usual limitation also identified, as in any study on poisoning by venomous animals, was that the results obtained cannot be extrapolated to the whole country, as it is a research carried out in only one service, extremely specialized and of reference, and the species of *Tityus* present in Brazil are diverse; and there are other variables that influence the severity and prognosis of these cases.

## CONCLUSION

The epidemiological profile found in this sample was a mean age of 36.05 years, mostly women, and non-occupational accidents ocurring in the urban area. The most frequently identified scorpion was the *Tityus serrulatus*. This study makes a contribution on the age groups at higher risk for the severity of scorpion stings. In the models analyzed, the risk factors found for moderate and severe classification were age group up to 22 years and previous care in other health services. We suggest the conduction of further studies to determine which age group should be considered a risk factor for the severity of scorpion stings. The age should be used as a predictive factor of severity in the clinical evaluation of patients stung by scorpions so as that adequate management of cases takes place.

## Data Availability

As recommended by *Ciência Aberta*, research data were entered in the Research Data Repository (REDU) at UNICAMP, under DOI number: https://doi.org/10.25824/redu/S42MWA
